# A new FTIR method for estimating the firing temperature of ceramic bronze-casting moulds from early China

**DOI:** 10.1038/s41598-021-82806-z

**Published:** 2021-02-08

**Authors:** Bichen Yan, Siran Liu, Matthew L. Chastain, Shugang Yang, Jianli Chen

**Affiliations:** 1 Institute for Cultural Heritage and History of Science and Technology, University of Science and Technology Beijing, Beijing, China; 2 Department of the History of Science, Tsinghua University, Beijing, China; 3 Department of Materials Science and Engineering, MIT, Cambridge, USA; 4 Stanford Archaeology Center, Stanford University, Stanford, USA; 5 Henan Provincial Institute of Cultural Heritage and Archaeology, Zhengzhou, China; 6 School of Archaeology and Museology, Peking University, Beijing, China

**Keywords:** Chemistry, Materials science

## Abstract

Intricate ceramic bronze-casting moulds are among the most significant archaeological remains found at Bronze Age metallurgical workshops in China. Firing temperature was presumably one of the most important technical factors in mould making. However, it has proven difficult to determine the firing temperatures of excavated moulds using existing analytical methods. This study establishes an innovative new method for using Fourier-transform infrared spectroscopy (FTIR) to estimate the firing temperature of clay-containing remains. The method is based on the finding that the infrared absorptivity of fired clay minerals, measured at the Si–O–Si stretching resonance band, is negatively correlated with firing temperature. Moulds and mould cores dating to the Early Shang period (sixteenth to fourteenth century BCE) are found to have been fired at extremely low temperatures—as low as 200–300 °C in many instances. These results provide critical new data for understanding the metallurgical technology of ancient China.

## Introduction

The production of bronze vessels was the most prominent handicraft industry in Bronze Age China (c. twentieth to fifth century BCE) and was deeply embedded in the ritual and political systems of the Shang and Zhou dynasties. This essential industry has received abundant scholarly attention, including ongoing debates over the location of metal sources^1,2^, distribution networks^3^ and casting techniques^4^. Researchers have also increasingly focused on the tools used in the bronze production process and, in particular, on the manufacture of the high-performance ceramic moulds that made possible the complex casting methods used in ancient China^5,6^. Understanding this mould-making technology is an essential component of the broader effort to understand diachronic change and regional variation in China’s metallurgical industries during the Bronze Age. The firing temperature of moulds deserves particular attention, as it is a key technical factor in mould making, capable of significantly influencing the performance of ceramic moulds and mould cores during the bronze-casting process.

However, using laboratory analyses to estimate the firing temperatures of excavated moulds and cores has been proved challenging. Initial attempts, based on measurement of moulds’ thermal expansion behavior, found that moulds were fired at 900–1050 °C^7^, with some scholars additionally claiming that moulds’ firing temperatures were deliberately kept between the decomposition temperature of calcite (850–900 °C) and the sintering temperature of typical clays (950–1000 °C)^8,9^. However, research has shown that the thermal expansion method used in these initial analyses may significantly overestimate the firing temperatures of low-fired ceramics—i.e., of ceramics fired below the sintering temperature of clay^10^. Recent investigations, using analytical methods better suited to low-fired ceramics, have found that moulds may have been fired at temperatures much lower than initially believed. Using a modified thermal expansion method, moulds and cores from the Western Zhou site of Zhouyuan (eleventh to eighth century BCE) and the Late Shang site of Xiaomintun (thirteenth to eleventh century BCE) were found to have been fired at 550–650 °C or lower^11,12^. Additional mould and core samples from Xiaomintun, analyzed using thermoluminescence, were found to have been fired at 600–700 °C^13^. Eastern Zhou-period moulds from Xinzheng (eighth-third century BCE) were determined to have been fired below 800 °C, due to the low degree of clay sintering observed by SEM imaging^5^. Finally, a large study of 72 moulds from Zhouyuan used Fourier-transform infrared spectroscopy (FTIR) to determine that almost all of the moulds had been fired at temperatures between 400 and 700 °C^14^. This latter study^14^ was the first to show that FTIR can be used to accurately estimate the firing temperature of ancient Chinese casting moulds, and it also demonstrated that, relative to alternative methods, FTIR is rapid, inexpensive, and requires only a tiny sample of artifact material.

In recent years, archaeologists have increasingly used FTIR analysis to study remains containing thermally altered clays^15^. Irreversible transformations that occur during the firing of clays and related silicate minerals can be identified in FTIR absorption spectra, making it possible to estimate the firing temperatures of ancient ceramics^16–18^ and of heated sediments^19–21^. Established FTIR-based approaches have generally involved first heating local sediments (or other representative raw materials) to various temperatures and then using these refired specimens as comparative standards to estimate the original firing temperatures of archaeological samples^22^. Thermal alteration appears most clearly in three regions of clays’ FTIR absorption spectra: the main Si–O–Si stretching vibration band (at wavenumbers around 1000–1100 cm^−1^), the Si–O–Al bending vibration band (510–580 cm^−1^), and the stretching vibration band of bound hydroxyl groups (3600–3700 cm^−1^). Changes to these three absorption bands—e.g., shifts in peak position or changes in peak shape—have therefore served in past FTIR-based studies as qualitative criteria used to estimate firing temperature^19,23^.

Nonetheless, estimating firing temperatures in this manner is made difficult by the fact that the position of the main Si–O–Si absorption peak—the primary temperature indicator in most FTIR studies—does not always change continuously with increasing firing temperature^19,24^. For many clays, the position of this peak (initially around 1030 cm^−1^) remains unaltered in the range of 500–800 °C but shifts abruptly to higher wavenumbers (1080–1090 cm^−1^) once heated over 800 °C^24^. Due to this behavior, established FTIR methods may in many cases produce firing temperature estimates too imprecise to be meaningful for addressing archaeological questions.

We suggest that this obstacle can be overcome by measuring temperature-dependent changes to the strength of infrared absorption in the main Si–O–Si stretching band—i.e., by measuring the height of the main Si–O–Si peak rather than its position. However, this approach must contend with a fundamental challenge: the intrinsic absorptivity of the sample material, which is the quantity relevant for estimating firing temperature, cannot be inferred directly from the peak height value measured in an absorption spectrum. This is because the absolute height of an absorption peak is determined not only by the sample’s intrinsic absorptivity but also by the measurement conditions—namely, the optical path length and the concentration of the sample, as described by the Beer–Lambert law (see below)—and these measurement conditions inevitably differ somewhat from sample to sample. A previous study of ancient Corinthian pottery circumvented this problem by measuring the ratio between the heights of two separate infrared absorption peaks within each spectrum^25^. The ratio was found to vary linearly with temperature, making it a useful indicator of firing temperature. However, that study’s methodology relies on compositional features specific to Corinthian clays and therefore cannot be applied to ceramics from other places.

**Figure 1:**
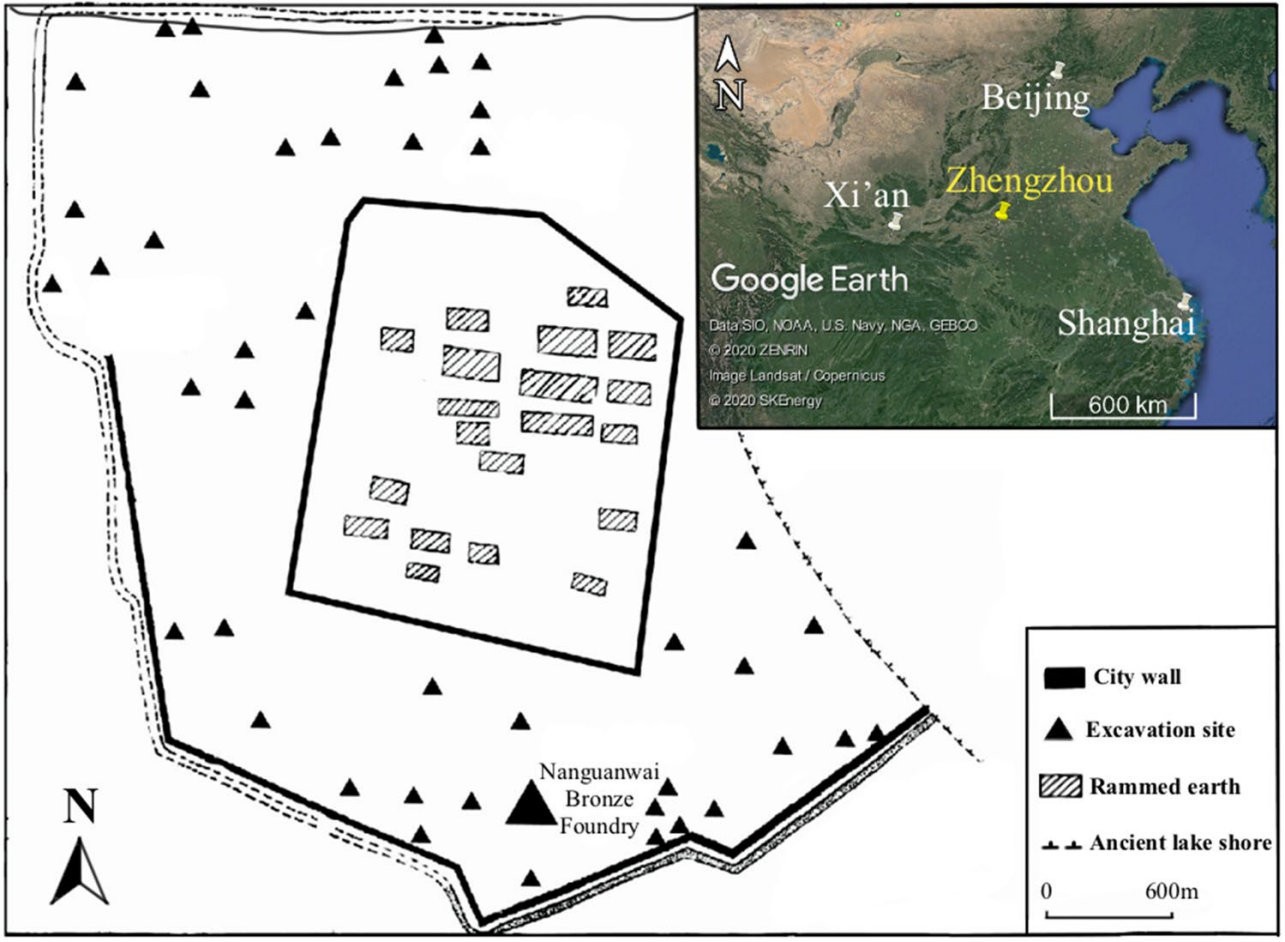
Map showing the location of Nanguanwai bronze foundry within the Zhengzhou Shang City archaeological site (the map was modified from^27^) and the location of Zhengzhou in north-central China. (the inset map was produced with Google Earth Pro 7.3.3.7786: https://www.google.com/intl/zh-TW_ALL/earth/versions/#download-pro, map data: Google, SKEnergy, ZENRIN).

In this present study, we develop the Absorptivity Ratio Method, a novel FTIR-based approach for estimating ceramic firing temperature. The method involves (1) adding a standard compound (potassium ferricyanide) to archaeological samples and (2) using this internal standard to quantify the intrinsic absorptivity of each archaeological sample within the region of the infrared spectrum corresponding to the main Si–O–Si stretching band. Irreversible, temperature-dependent changes in absorptivity can thereby be detected accurately and used to infer firing temperature. We use this method to estimate the firing temperatures of bronze-casting moulds and cores, dating to the Early Shang period (sixteenth to fourteenth century BCE), excavated from the Nanguanwai bronze workshop at the Zhengzhou Shang City site, a capital city of the Early Shang dynasty (Fig. 1)^26^. The results help to resolve the ongoing controversy (discussed above) concerning the firing temperature of bronze-casting moulds in ancient China and, furthermore, contribute significantly to our understanding of the technological style of bronze casting during the Early Shang period, a seminal era for early metallurgy in China.

### The Absorptivity Ratio Method: theory and proof of concept

Infrared absorption is governed by the Beer–Lambert law:

$$A\left( \lambda \right) = a(\lambda ) \cdot c \cdot b$$

where *A*(*λ*) is the measured absorbance value (i.e., the peak height) of the analyte at wavenumber *λ*; *a*(*λ*) is the absorptivity coefficient at wavenumber *λ*, which is determined by the molecular structure of the analyte; *c* is the concentration of the analyte within the prepared sample; and *b* is the optical path length (equal to the thickness of the KBr sample pellet for samples prepared in this manner).

The value of *a*(*λ*), the absorptivity coefficient at a given wavenumber λ, is related to the polarity of the chemical bonds and groups present in a material. For some substances, like clay minerals, these aspects of molecular structure change progressively with increasing temperature, resulting in corresponding changes in infrared absorption^19^. In clays, changes to the main Si–O–Si stretching vibration band (around 1030 cm^−1^) can begin at temperatures as low as 200 °C. At 400–600 °C, this absorption band becomes broadened. And, between 700 and 1000 °C, it shifts toward higher wavenumbers. These changes reflect a gradual breakdown of clays’ ordered molecular structure, including: condensation of Si–O tetrahedra, breaking of Si–O–Al bonds, and loss of hydroxyl (-OH) groups^28^. These same changes also tend to reduce the strength of infrared absorption within the main Si–O–Si stretching band, making absorptivity, *a*(*λ*), a potentially useful indicator of firing temperature.

**Figure 2:**
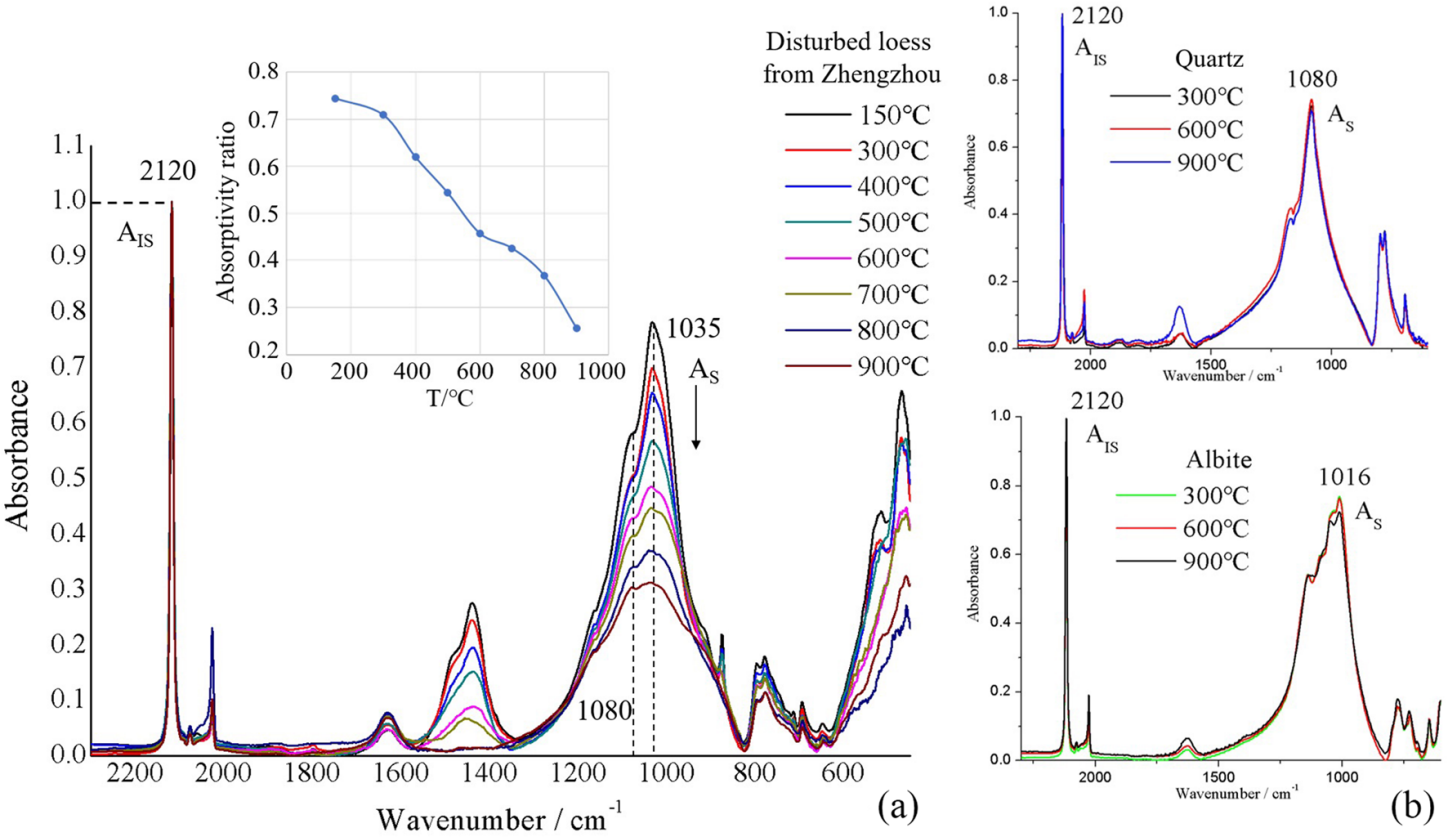
(**a**) Infrared spectra of Zhengzhou soil heated to various temperatures and mixed with potassium ferricyanide as an internal standard. Mass ratio (m_IS_/m_S_) is equal to one for all samples. All spectra are normalized to the peak absorbance value of the internal standard (A_IS_, located at wavenumber 2120 cm^−1^). The normalized height of the soil samples’ main absorption peak (at 1035 cm^−1^) decreases steadily as the firing temperature is increased from 150 to 900 °C (see inset plot); (**b**) Infrared spectra of quartz and of albite feldspar heated to various temperatures and mixed with potassium ferricyanide as an internal standard. For both quartz and albite, the normalized height of the main absorption peak (at 1080 cm^−1^ and 1016 cm^−1^, respectively) remains constant as firing temperature is increased.

Due to the Beer–Lambert law, direct measurement of the absorptivity, *a*(*λ*), of a material requires precise control over sample concentration and optical path length, making such measurements impractical in most cases. However, established methods, such as the internal standard method^29,30^ and the band ratio method^31,32^, allow absorptivity to be quantified independently of sample concentration and optical path length. We use an internal standard method, in which the absorptivity of clay is measured relative to that of an internal standard compound added to the sample. Potassium ferricyanide (K_3_[Fe(CN)_6_]) was chosen as the internal standard, as it has no band overlap with clay minerals (Fig. 2) or with other minerals present in the casting mould samples.

Applying the Beer–Lambert law to the internal standard and the sample yields the following equations:

1 $$A_{IS} \left( {\lambda_{1} } \right) = \, a_{IS} (\lambda_{1} ) \cdot c_{IS} \cdot b$$

2 $$A_{S} \left( {\lambda_{2} } \right) = \, a_{S} (\lambda_{2} ) \cdot c_{S} \cdot b$$

where *A*_*IS*_(*λ*_*1*_) and *A*_*S*_(*λ*_*2*_) are the measured absorbance values (peak heights) of the internal standard and the sample at their respective main peaks and *a*_*IS*_(*λ*_*1*_) and *a*_*S*_(*λ*_*2*_) are the corresponding absorptivity coefficients. Dividing Eq. (2) by Eq. (1) gives:

$$a_{S} (\lambda_{2} )/a_{IS} (\lambda_{1} ) = c_{IS} /c_{S} \cdot A_{S} (\lambda_{2} )/A_{IS} (\lambda_{1} )$$

And, since the ratio of the concentrations of the internal standard and the sample, *c*_*IS*_*/c*_*S*_, is equal to their mass ratio, *m*_*IS*_*/m*_*S*_, the following formula also holds:

3 $$a_{S} (\lambda_{2} )/a_{IS} (\lambda_{1} ) = m_{IS} /m_{S} \cdot A_{S} \left( {\lambda_{2} } \right)/A_{IS} \left( {\lambda_{1} } \right)$$

Thus, the *absorptivity ratio*—defined as *a*_*S*_(*λ*_*2*_)*/a*_*IS*_(*λ*_*1*_), a normalized measure of a sample’s absorptivity coefficient—can be calculated by simply measuring the peak heights of the sample and the internal standard, given that the two substances were mixed at a known mass ratio.

A proof-of-concept experiment was undertaken to confirm the validity of absorptivity ratio as an indicator of firing temperature. Figure 2a shows infrared spectra of Zhengzhou soil samples heated to various temperatures. All spectra are normalized to the height of the internal standard’s main peak. As firing temperature is increased from 150 to 900 °C, the normalized height of the soil samples’ main absorption peak (around 1035 cm^−1^) decreases in a consistent and approximately linear fashion. This decrease can be attributed mainly to structural changes within clay minerals, as other silicates present in the soil (quartz and feldspar) show negligible temperature-related change. These results demonstrate that absorptivity ratio, which is equal to the normalized height of the soil samples’ main peak, is related to firing temperature in a clear and predictable way and can therefore be expected to serve as an effective indicator of the firing temperature of archaeological samples.

## Results

### Composition and microstructure

**Figure 3:**
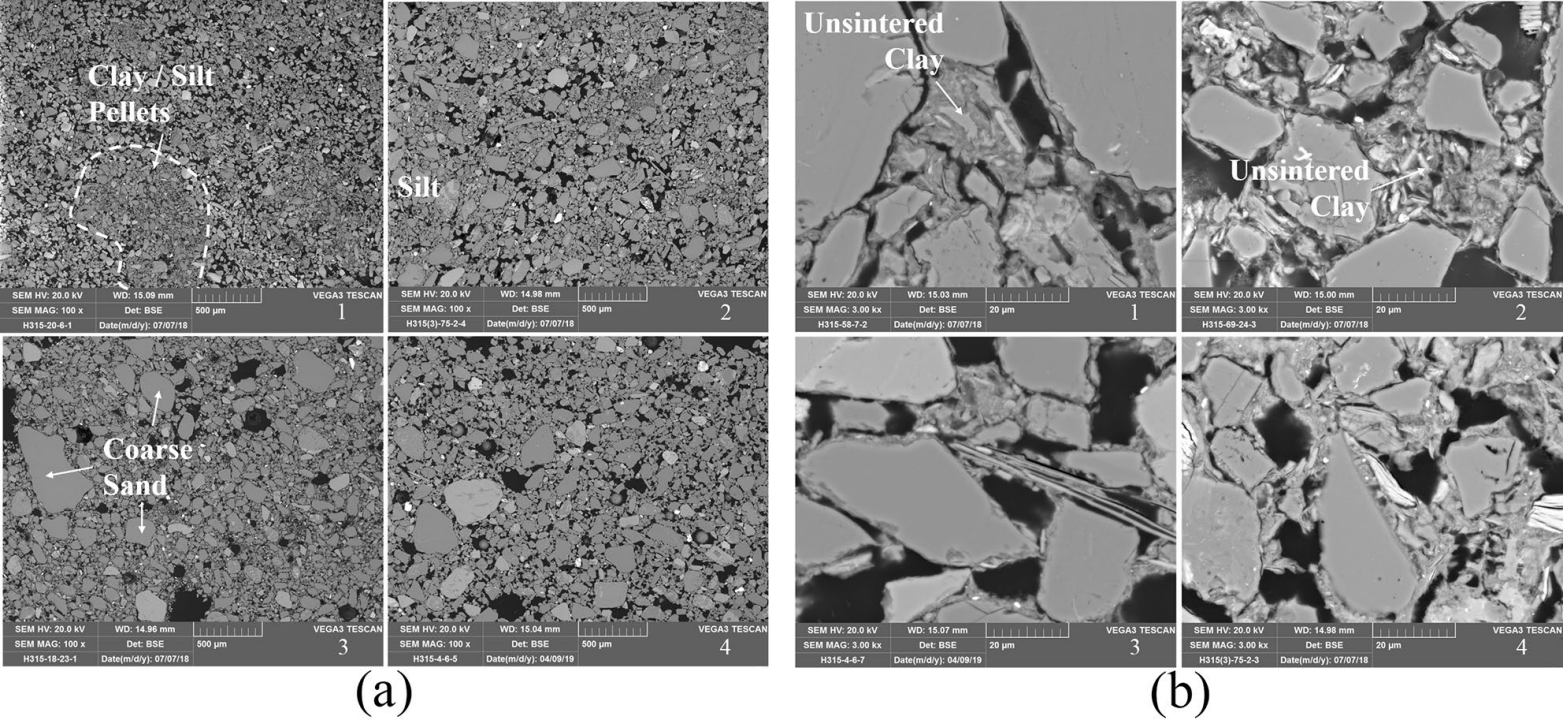
(**a**) Low-magnification SEM BSE micrographs of mould and core samples. (1) H315:20–6, mould; (2) H315③:75–2, mould; (3) H315:18–23, core; (4) H315:4–6, core. All × 100 magnification; (**b**) High-magnification SEM BSE micrographs of mould and core samples. (1) H315:58–7, mould; (2) H315:69–24, mould; (3) H315:4–6, core; (4) H315③:75–2, core. All × 3000 magnification.

The mineralogical compositions of two moulds were determined by XRD and are shown in Supplementary Table S1 online. The bulk elemental compositions for all moulds and cores were obtained by SEM–EDS analysis and are presented in Supplementary Table S2 online. The microstructures of moulds and cores were revealed by BSE imaging (Fig. 3). The mould and core samples show little variation in their chemical compositions. Silica and alumina content are typically between 70–80 and 10–16%, respectively. The mean silica content of cores (77.9%) is slightly higher than that of moulds (73.6%). The iron content of both moulds and cores is between 3 and 6%. Calcium content is generally between 1 and 3%, indicating that little calcite is present in the fabric. XRD analysis of two moulds finds that the major mineralogical phases present are quartz, feldspars (albite and microcline), and clay minerals (illite, kaolinite, and smectite), without any significant amount of calcite. The total content of clay minerals is less than 15%, and the primary mineral components of the clay are illite and illite–smectite mixed-layer minerals. The sharp illite peaks indicate that both samples were fired at relatively low temperature^19^.

The microstructures of moulds and cores share similar features. The fabrics of these samples are highly porous and consist mainly of silt and sand particles, which are held together by a lesser proportion of clay matrix. Compared to moulds and cores from the Late Shang period, the Early Shang samples studied here contain a larger proportion of clay and contain silt/sand particles with a more poorly sorted size distribution^6^. The clay matrix in these moulds is distributed unevenly throughout the fabric, with clay-rich pellets up to thousands of microns in size observed occasionally (Fig. 3a). Clay particles within the matrix generally exhibit a low degree of sintering, again suggesting a low firing temperature (Fig. 3b).

### Firing temperature estimation by FTIR qualitative analysis

**Figure 4:**
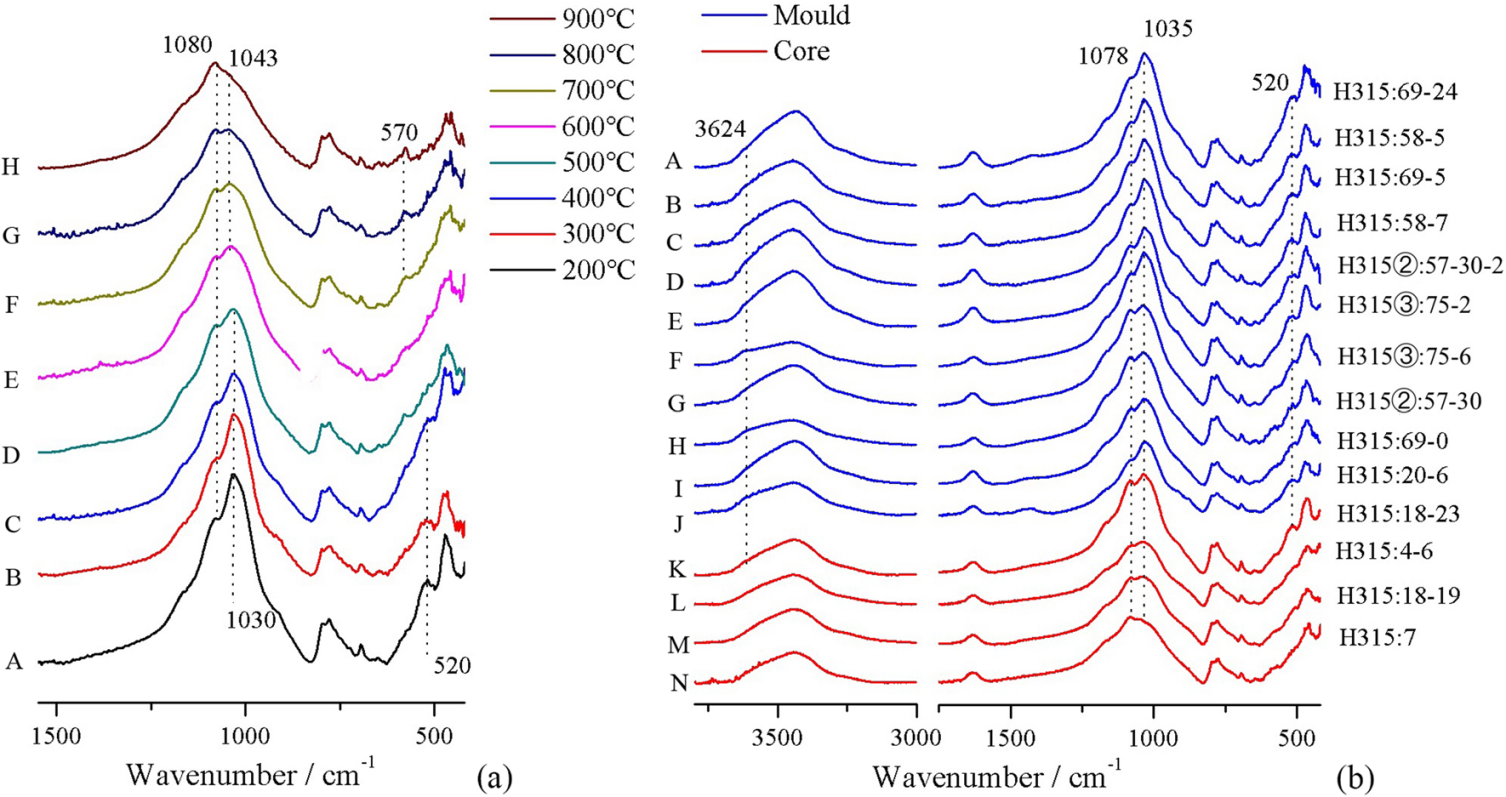
(**a**) Infrared spectra of mould *H315:58–7* heated to different temperatures. (A) Material heated at 200 °C. (B-C) Material heated at 300 °C and 400 °C. No major infrared spectral changes are detected. (D-E) Material heated at 500 °C and 600 °C. The Al–O–Si absorption at 520 cm^−1^ is now absent. The Si–O–Si absorption peak at 1033 cm^−1^ decreases and shifts from 1033 cm^−1^ to 1043 cm^−1^. (F) Material heated at 700 °C. The quartz absorption shoulder at 1080 cm^−1^ increases. A characteristic AlO_4_ tetrahedra absorption peak appears at 570 cm^−1^, likely related to dehydroxylation of Al[O(OH)]_6_ octahedra. (G-H) Material heated at 800 °C and 900 °C. The Si–O–Si absorption at 1043 cm^−1^ decreases further. (**b**) Infrared spectra of all mould and core samples. These spectra show samples that have not been heated in the laboratory. (A–K) All moulds and one core display clay and quartz peaks without signs of thermal alteration. Note the clay shoulder at 3624 cm^−1^ and the major clay absorption peaks at 1035 cm^−1^ and 520 cm^−1^. (L–N) The remaining three cores do show indicators of thermal alteration. In these samples, the clay peak at 520 cm^−1^ has disappeared and the main clay Si–O–Si absorption peak at 1035 cm^−1^ has decreased and shifted toward higher wavenumbers.

Figure 4a shows the FTIR absorption spectra of one mould sample (H315:58-7) after being reheated to various temperatures. At 500 °C and above, the 520 cm^−1^ absorption band disappears and the 1035 cm^−1^ band becomes weaker. At 700 °C and above, an Al-O tetrahedron absorption band appears at 570 cm^−1^. Since all mould and core samples are generally similar in terms of composition, these changes observed in sample H315:58-7 can be used as reference points to infer the original firing temperatures of the other samples. Figure 4b shows infrared absorption spectra for all samples. A relatively low firing temperature can be determined qualitatively for all moulds and one core (H315:18-23) due to their strong Si–O–Si stretching vibration band at 1035 cm^−1^, together with the presence of a Si–O–Al bending vibration band at 520 cm^−1^ and a weak Al–OH stretching band at 3642 cm^−1^. Infrared spectra of the remaining three cores show features consistent with higher firing temperatures: a weaker 1035 cm^−1^ band, along with the absence of 520 cm^−1^ and 3642 cm^−1^ bands. The 570 cm^−1^ band is not present in any of the samples. Thus, the original firing temperatures of all moulds and of core H315:18-23 were evidently below 500 °C, while the other three cores each appear to have been fired to between 500 and 700 °C.

### Firing temperature estimation by the Absorptivity Ratio Method

**Figure 5:**
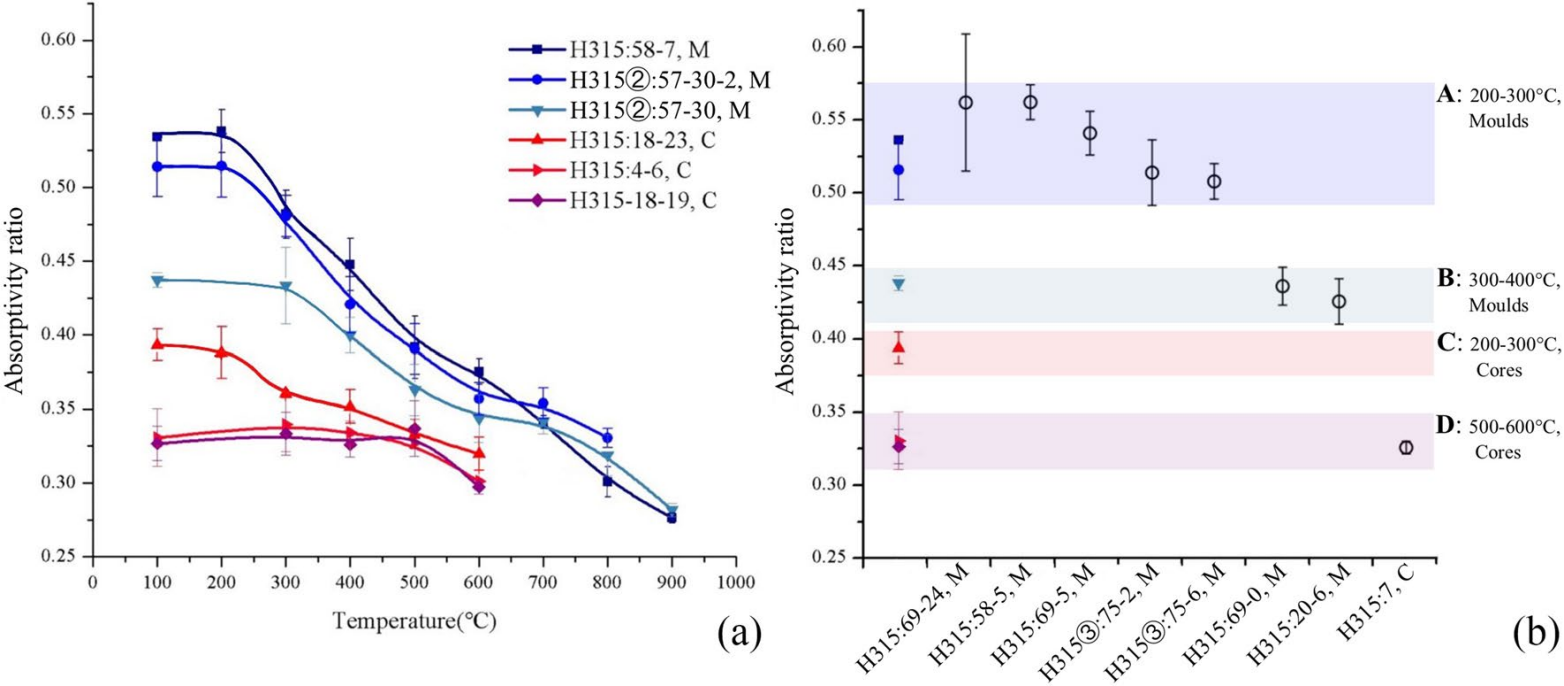
(**a**) Firing temperature estimation via the refiring approach. Change in absorptivity ratio with increasing firing temperature is plotted for six artifacts subjected to laboratory refiring. The top three curves are moulds (M), and the bottom three are cores (C). Error bars represent the standard deviation among three samples measured per artifact; (**b**) Firing temperature estimation via the calibration curve approach. Absorptivity ratio values are plotted for the six artifacts analyzed using the refiring approach (colored data points; shapes/colors correspond to those used in (**a**)) and for the eight artifacts not subjected to refiring (unfilled circles). Four clusters (A-D) are identified and correlated with firing temperature estimates made using the refiring approach.

The average absorptivity ratio values for all artifacts are listed in Supplementary Table S3 online. In order to correlate these measured values to the artifacts’ firing temperatures, six selected artifacts (three moulds and three cores) were analyzed by the refiring approach (RA). This involved refiring samples of each artifact at various temperatures and observing the resulting changes in the absorptivity ratio (Fig. 5a). At low refiring temperatures, each sample’s absorptivity ratio remains constant. However, above a threshold temperature, the absorptivity ratio begins to decline steadily with increasing refiring temperature. This threshold indicates the point at which the refiring temperature first exceeds an artifact’s original firing temperature. For example, the absorptivity ratio versus temperature curve of mould H315:58-7 begins to slope downward between 200 and 300 °C, implying an original firing temperature within that range.

The six artifacts analyzed by the refiring approach establish a calibration curve that correlates an artifact’s (non-refired) absorptivity ratio to its original firing temperature. Using this calibration curve approach (CCA), firing temperature estimates can be made for the remaining eight artifacts (seven moulds and one core) without the use of further refiring. As shown in Fig. 5b, artifacts’ absorptivity ratio values fall into four clusters, labeled A-D: cluster A corresponds to moulds fired between 200 and 300 °C, cluster D to cores fired between 500 and 600 °C, etc. Note that separate calibration curves are required for moulds and for cores, as the material used to make cores exhibits significantly lower absorptivity across the range of firing temperatures studied.

Through this combination of the refiring approach and the calibration curve approach, it was found that, out of ten total moulds, seven were originally fired at 200–300 °C and three at 300–400 °C. Additionally, out of four total cores, one was fired at 200–300 °C and three at 500–600 °C. For every artifact, these Absorptivity Ratio Method findings are consistent with the independent estimates of firing temperature made via FTIR qualitative analysis (see Supplementary Table S3 online).

## Discussion

Absorptivity ratio is related to both the firing temperature and the clay mineral content of a sample. Thus, combining infrared analysis with compositional analysis is necessary for the Absorptivity Ratio Method. For an initial group of samples analyzed by this method, absorptivity ratio versus temperature curves must be established by refiring each sample to various temperatures. In this refiring approach (RA), a sample’s original firing temperature can be identified by the presence of a change in the slope of the curve. Subsequently, the calibration curve approach (CCA) can be used to estimate the firing temperatures of additional samples without the need for further refiring. This entails comparing the absorptivity ratios of non-refired samples to the absorptivity ratio values of samples whose firing temperatures were determined via the RA. While the CCA admittedly estimates firing temperature with greater uncertainty, it is also far more efficient that the RA, making it feasible to analyze large artifact assemblages. Out of fourteen artifacts in this study, the RA was applied to six and the CCA to eight. When using the CCA, moulds and cores required separate calibration curves. This was unexpected, given the similarity in elemental composition among all artifacts. While the difference in behavior between moulds and cores may be due to differences in clay mineralogy, it was not possible to confirm this directly, as no core samples reached the minimum mass needed for XRD quantitative analysis. Ultimately, the overall validity of the Absorptivity Ratio Method results is corroborated by two additional analyses. First, the artifact samples behaved as predicted by the proof-of-concept experiment. Second, the Absorptivity Ratio Method results agreed strongly with the results of an independent, qualitative FTIR analysis.

The Absorptivity Ratio Method offers several advantages over alternative methods. Many approaches to firing-temperature estimation—including thermoluminescence^13,33^, thermal expansion^10^, and magnetic susceptibility methods^34,35^—require that every individual sample be refired to a range of different temperatures, thus making it impractical to apply these methods to artifact assemblages of any significant size. FTIR is also more practical and economical than the instruments used for competing methods. FTIR spectrometers are comparatively inexpensive and robust (and even field portable in some cases). Sample preparation and analysis takes minutes and consumes just fractions of a gram of sample material. Additionally, the Absorptivity Ratio Method involves only the analysis of artifact samples. Unlike many other FTIR-based approaches, it does not require identifying and collecting period-authentic clays or other raw materials.

In this study, the Absorptivity Ratio Method was used to estimate the original firing temperatures of fourteen total mould and core samples. The estimated firing temperature values—between 200 and 400 °C for all ten moulds—are lower than those reported by any previous study of ancient casting moulds from China. Several mould-firing kilns from the Shang and Zhou periods have been compared to pottery kilns from the same era^36^. The structures of the mould-firing kilns are simpler and less standardized, suggesting they may have operated at lower temperatures than pottery kilns. And, in some cases, moulds might not have been fired in kilns at all. Researchers have suggested that, during the Late Shang period, moulds may have been simply dried in pits using an open fire^12^. Why would producers have chosen to fire their casting moulds at such low temperatures? It is instructive to observe that *The Pirotechnia of Vannoccio Biringuccio*, a sixteenth-century CE European treatise on metallurgy, describes several methods for baking moulds without kilns, emphasizing that firing should be done slowly and with a low flame in order to prevent cracking in the moulds^37^. Modern research shows that high porosity, high sand content, and low firing temperature can together improve resistance to thermal shock, as voids and particles serve to arrest the growth of cracks^38^. Several previous studies have suggested that the use of specialized, low-clay, high-silt mould materials developed indigenously in China during the Early Bronze Age^6,39,40^. It appears likely that deliberate low-temperature firing of moulds was also an essential part of the mould-making technological style of the Shang people. Evidence from bronze workshops at Anyang demonstrates the continuation and further development of this mould-making tradition into the Late Shang period^12,13^.

**Figure 6:**
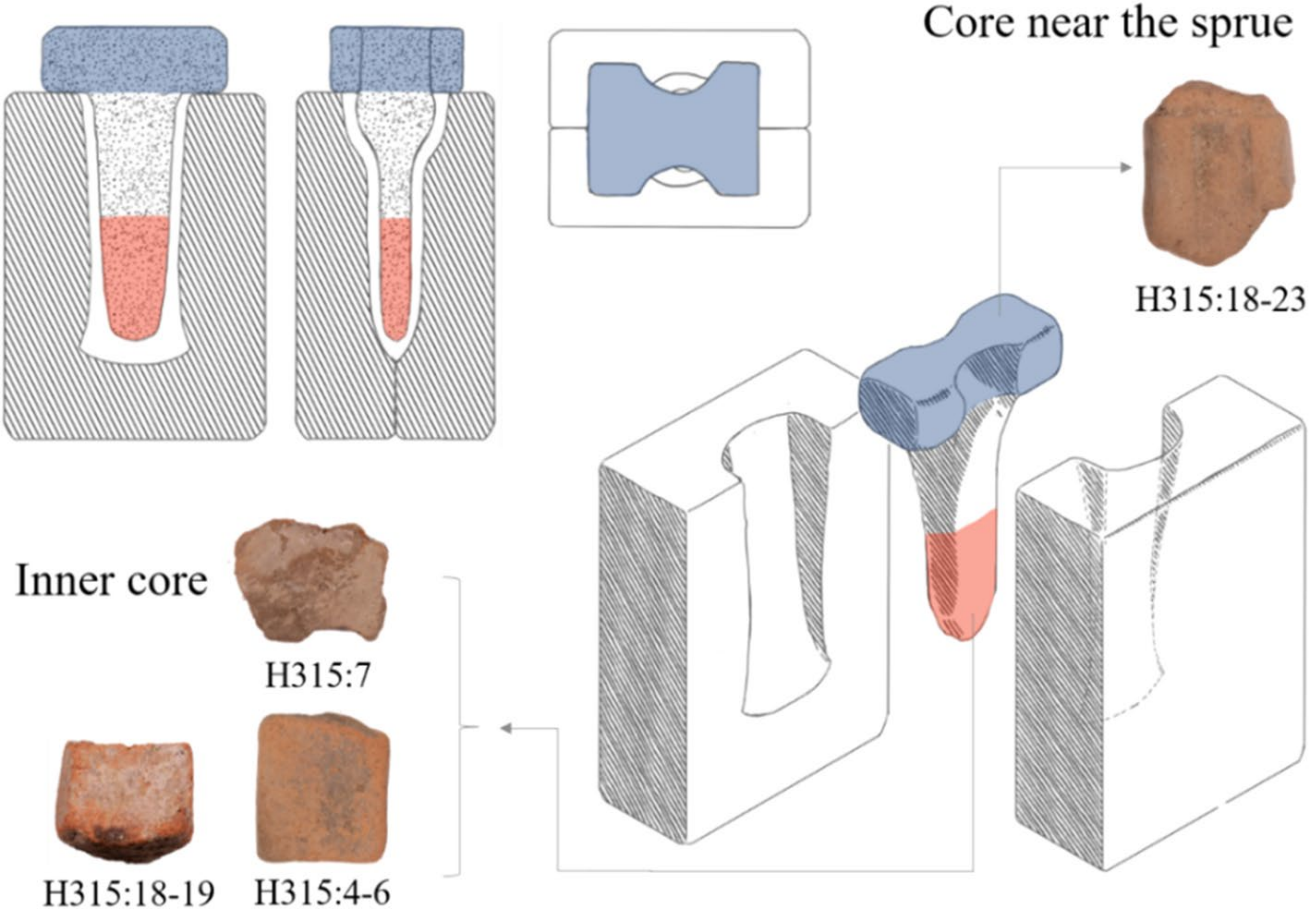
Illustration of a ceramic mould assembly for casting a socketed bronze adze. The core fragments analyzed in this study come from different regions of the core, as indicated.

Meanwhile, we find that most of the cores analyzed (three out of four) were fired at temperatures 200–300 °C higher than those used to fire moulds. It may be the case that moulds and cores were fired using different firing practices, but this issue still requires further investigation. Another possible explanation is that the apparent firing temperatures of the cores were influenced by exposure to hot metal during the casting process, a phenomenon noted by other researchers^13^. Most of the core artifacts in this study are fragments from the inner region of adze-mould cores, and therefore would have been surrounded by molten bronze for an extended duration during casting (Fig. 6). The only exception is core H315:18-23, which comes from near the sprue and was thus in contact with molten metal for a much shorter duration. Therefore, it may not be a coincidence that the estimated firing temperatures of the three inner core fragments are higher than those of core H315:18-23 and of all ten moulds.

These results provide important new data for understanding the development and regional variability of Bronze Age mould-making technology in China. The extremely low firing temperatures found in this study suggest that low-temperature firing may have been an essential feature of the technological style of Shang mould making from at least as early as the Erligang period. The use of low-fired moulds, made from highly porous, silty materials, appears to have enabled the Shang to cast the extraordinarily complex and high-quality bronzes for which they are celebrated today.

## Methods

### Samples

All samples examined in this study were excavated from the Nanguanwai bronze foundry, a part of the Zhengzhou Shang City site located outside the site’s southern city wall. Excavation at Nanguanwai began in 1954, unearthing a total area of 25,000 square meters of the bronze foundry site. Bronze-casting activities were carried out at the site from the Lower Erligang II period to the Upper Erligang I period (mid-fifteenth to mid-fourteenth century BCE). A significant quantity of metallurgical remains was unearthed, including copper-bearing minerals, slags, furnace and crucible fragments, and small bronze artefacts, as well as hundreds of thousands of ceramic casting moulds and casting-mould cores^26^.

This study examines ten mould fragments and four core fragments collected from ash pit H315 at the Nanguanwai bronze foundry (see Supplementary Fig. S1 online). This pit is dated to the Upper Erligang I period (fourteenth century BCE) based on its stratigraphy and on the typology of pottery sherds found in the pit^26^. It is unclear what types of bronze products the ten mould samples were used to cast, as the samples are all small and fragmentary. The cores were used to cast small, socketed bronze tools, such as adzes or chisels (Fig. 6). Additionally, a sample of local Zhengzhou soil was collected for use in a proof-of-concept firing experiment. This soil was collected from undisturbed strata and was typical secondary deposited loess without significant inclusions.

### Characterization of composition and microstructure

A Tescan Vega III scanning electron microscope (SEM) equipped with a Bruker XFLASH6|10 energy dispersive spectrometer (EDS) was employed for electron imaging and elemental analysis. All mould and core samples were subjected to these analyses. Being too fragile for cutting and polishing without treatment, the samples were initially consolidated with epoxy resin. All the samples were then mounted as blocks, ground, polished with 0.25 micron diamond paste, and carbon coated prior to SEM analysis. The acceleration voltage was set at 20 kV, and the working distance was kept at roughly 15 mm. The backscattered electron (BSE) imaging mode was used to study the microstructure of the samples. Bulk elemental composition was determined for each sample by averaging EDS analyses of three randomly selected 870 μm by 1270 μm areas. Considering the variable beam intensity of the SEM system and the porosity of the samples, all elemental data have been normalized to 100% and should be taken as semi-quantitative only.

X-ray diffraction (XRD) analysis was used to determine the mineralogical composition of mould samples H315:58-5 and H315:57-30. Analysis was carried out using a Rigaku D/Max-RB X-ray diffractometer with Cu K-alpha radiation. Powdered samples were measured for a range of two-theta values from 3° to 50°, at a rate of 2° per minute, and with a sampling interval of 0.02°. The operating voltage was 30 to 45 kV, and the operating current was 20 to 100 mA. Sample preparation and analysis followed a standard procedure, which required at least 10 g of sample^41^. The relative content of non-clay minerals was measured by the peak area ratio of specific peaks between mineral phases and internal standard Al_2_O_3_. The height and intensity of diffraction peaks of non-clay mineral phases were obtained from three XRD patterns of each sample. Clay-sized particles (under 10 μm diameter) were extracted by Stokes’ law sedimentation, and the total content of clay was determined by weighing. Quantitative analysis of clay minerals required the preparation of three different clay samples: an untreated sample, an ethylene glycol treated sample, and a thermally treated sample. Ethylene glycol treatment was used to distinguish swelling minerals and non-swelling minerals, while thermal treatment was used to determine the change in the interplanar spacing (d) of minerals after dehydration.

### Estimation of firing temperatures

For every mould and core artifact, firing temperature was estimated using two independent FTIR-based methods. First, FTIR qualitative analysis made estimates based on the presence, position, and shape of specific infrared absorption peaks associated with clay minerals. Second, the Absorptivity Ratio Method made estimates by quantifying the infrared absorptivity of clay minerals via the use of an internal standard. All FTIR measurements were performed in transmission mode using a Thermo Fischer Scientific Nicolet iS5 FTIR spectrometer. Absorption spectra were collected in the mid-IR region (wavenumbers between 4000 and 400 cm^−1^) with a spectral resolution of 4 cm^−1^ and 16 scans per sample. Infrared spectral data were analyzed using Thermo Scientific OMNIC software.

For FTIR qualitative analysis, sample preparation followed a standard procedure using potassium bromide (KBr) pellets^42^. Estimation of firing temperatures was based on criteria given in references 19 and 23. For the Absorptivity Ratio Method, artifact samples were combined with potassium ferricyanide (K_3_Fe(CN)_6_) as an internal standard. 0.2000 g of evenly ground sample material was mixed with 0.2000 g of potassium ferricyanide in an agate mortar and pestle. Masses were measured to within ± 0.0010 g using an analytical balance with a precision of 0.0001 g. 1–2 mg of mixed sample was then combined with 100–200 mg of KBr and prepared as a pellet. Each mould and core was sampled and analyzed three times. To ensure that heterogeneity within artifacts did not cause erroneous results, sample material was collected from a region extending from the surface of each artifact into the interior. Thus, each FTIR sample represents the full thickness of the sampled artifact.

In collected FTIR absorption spectra, peak height was measured by the baseline method. Baseline correction is necessary to make the baseline horizontal and to measure peak height precisely. The calibration wavenumbers were set as 3860, 3075, 2200, 1940, 1730, 1570, 960, 665 cm^−1^. Peak height was measured for the main potassium ferricyanide peak at 2120 cm^−1^ and for the main sample peak at 1030 cm^−1^; datum points were set as 2200 to 1960 cm^−1^ and 1550 to 830 cm^−1^ (Fig. 3). Measured absorptivity values (peak heights) were then substituted into Formula (3) to calculate the absorptivity ratio. Each sample was measured three times in order to calculate the mean absorptivity ratio and the relative measurement error (coefficient of variation).

Two variants of the Absorptivity Ratio Method were used: the refiring approach (RA) and the calibration curve approach (CCA). For the six artifacts analyzed by the refiring approach, samples of artifact material were refired to a range of temperatures that increased from 200 to 900 °C in 100 °C increments. Samples were fired in a corundum crucible for 3 h in a muffle furnace with an air atmosphere. The refired samples were then cooled to ambient temperature prior to FTIR analysis. For the eight artifacts analyzed by the calibration curve approach, samples were not refired before FTIR analysis.

## Supplementary Information

Supplementary Information.

## Data Availability

The datasets generated and analysed as part of this study are available from the corresponding author on reasonable request.
