# Supplementary material for: A new FTIR method for estimating the firing temperature of ceramic bronze-casting moulds from early China
Source: Sci Rep. 2021 Feb 8;11:3316. doi: 10.1038/s41598-021-82806-z (PMC7870837; doi:10.1038/s41598-021-82806-z)
Supplement: Supplementary file 1 — Supplementary Information. [file 41598_2021_82806_MOESM1_ESM.pdf]

A new FTIR method for estimating the firing temperature of ceramic bronze-casting moulds from early China

Bichen Yan [1,2], Siran Liu[1*], Chastain Matthew[3], Shugang Yang[4], Jianli Chen[5]

1 Institute for Cultural Heritage and History of Science & Technology, University of Science and Technology Beijing
2 Department of the History of Science, Tsinghua University
3 Department of Materials Science and Engineering, MIT
4 Henan Provincial Institute of Cultural Heritage and Archaeology
5 School of Archaeology and Museology, Peking University
* Email: siran.liu@ustb.edu.cn

Supplementary Information

Supplementary Table S1. Mineralogical composition of two mould samples.

All phases				
**Sample**	**Quartz**	**Albite**	**Microcline**	**Clay**
**H315②:57-30**	57%	20%	9%	14%
**H315:58-5**	46%	27%	12%	15%

Clay minerals						
**Sample**	**Clay mineral composition**					**Mixed-layer ratio (S%)**
	**It/S**	**It**	**Kao**	**It/V**	**Pa**	**It/S**
**H315②:57-30**	72%	25%	3%	-	-	40%
**H315:58-5**	52%	40%	5%	2%	1%	40%

It: Illite; Kao: Kaolinite; V: Vermiculite; Pa: Palygorskite; S: Smectite

Supplementary Table S2. Chemical composition of all mould and core samples.

Sample	Type	Na_2O	MgO	Al_2O_3	SiO_2	K_2O	CaO	FeO
**H315③:75-6**	mould	1.6	1.0	12.3	75.3	2.2	2.1	4.5
**H315③:75-2**	mould	1.7	1.1	13.2	74.9	2.9	1.5	4.4
**H315:20-6**	mould	1.1	Bdl	12.3	74.6	2.2	2.5	6.3
**H315:69-0**	mould	2.1	1.4	13.4	72.1	3.6	2.1	5.0
**H315:69-24**	mould	2.0	1.1	12.5	73.4	3.0	3.4	4.3
**H315:69-5**	mould	1.7	1.1	14.9	73.4	3.5	1.1	3.8
**H315:58-5**	mould	2.0	1.1	15.7	71.2	3.6	1.4	4.7
**H315:58-7**	mould	1.9	1.2	14.5	73.1	3.3	1.4	4.3
**H315②:57-30**	mould	1.7	0.9	11.3	75.1	1.7	2.7	4.3
**H315②:57-30-2**	mould	2.0	1.0	16.0	72.4	3.1	1.1	3.7
**Average of moulds**		1.8	1.1	13.6	73.6	2.91	1.9	4.5
**H315:18-19**	core	1.0	Bdl	10.3	80.6	2.2	1.2	4.7
**H315:7**	core	2.1	Bdl	13.2	75.6	2.7	1.4	4.5
**H315:18-23**	core	1.7	Bdl	10.7	80.8	2.5	1.3	2.8
**H315:4-6**	core	2.2	1.0	13.7	74.7	2.9	1.6	3.5
**Average of cores**		1.8	0.3	12.0	77.9	2.6	1.4	3.9

Supplementary Table S3. Estimated firing temperature ranges for all mould and core samples.

Sample	Type	FTIR qualitative analysis		FTIR quantitative analysis (Absorptivity Ratio Method)			
		Observed FTIR absorption bands (cm^{-1})	Firing temperature (ºC)	Average absorptivity ratio (a_S/a_{IS})	Coefficient of variation	Firing temperature (ºC)	Estimation approach
H315:69-24	mould	3624, 1035, 520	＜500	0.5616	8.33%	200-300	CCA
H315:58-5	mould	3624, 1035, 520	＜500	0.5614	2.12%	200-300	CCA
H315:69-5	mould	3624, 1035, 520	＜500	0.5411	2.76%	200-300	CCA
H315:58-7	mould	3624, 1035, 520	＜500	0.5344	0.27%	200-300	RA
H315②:57-30-2	mould	3624, 1035, 520	＜500	0.5139	3.96%	200-300	RA
H315③:75-2	mould	3624, 1035, 520	＜500	0.5133	4.36%	200-300	CCA
H315③:75-6	mould	3624, 1035, 520	＜500	0.5073	2.39%	200-300	CCA
H315②:57-30	mould	3624, 1035, 520	＜500	0.4373	1.13%	300-400	RA
H315:69-0	mould	3624, 1035, 520	＜500	0.4362	2.96%	300-400	CCA
H315:20-6	mould	3624, 1035, 520	＜500	0.4262	3.64%	300-400	CCA
H315:18-23	core	3624, 1035, 520	＜500	0.3934	1.19%	200-300	RA
H315:4-6	core	1078, 1035	500-700	0.3307	5.91%	500-600	RA
H315:18-19	core	1078, 1035	500-700	0.3268	3.58%	500-600	RA
H315:7	core	1078	500-700	0.3259	1.32%	500-600	CCA

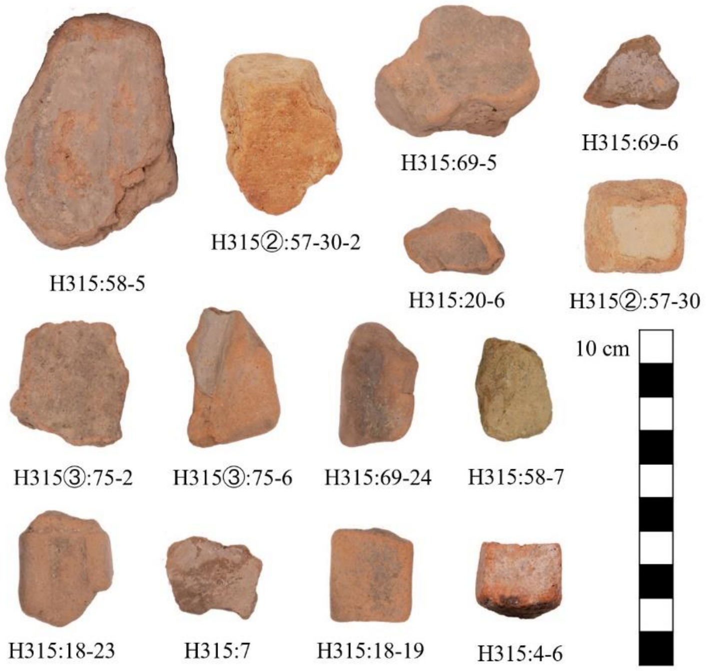

Supplementary Figure S1. All bronze-casting mould and core artifacts analyzed in this study.
